# Complex Network-Driven View of Genomic Mechanisms Underlying Parkinson's Disease: Analyses in Dorsal Motor Vagal Nucleus, Locus Coeruleus, and Substantia Nigra

**DOI:** 10.1155/2014/543673

**Published:** 2014-11-26

**Authors:** Beatriz Raposo Corradini, Priscila Iamashita, Edilaine Tampellini, José Marcelo Farfel, Lea Tenenholz Grinberg, Carlos Alberto Moreira-Filho

**Affiliations:** ^1^Department of Pediatrics, Faculdade de Medicina da USP (FMUSP), Avenida Dr. Enéas Carvalho Aguiar 647, 5 Andar, 05403-900 São Paulo, SP, Brazil; ^2^Brazilian Aging Brain Study Group (BEHEEC), LIM 22, FMUSP, 01246-903 São Paulo, SP, Brazil; ^3^Hospital Israelita Albert Einstein, 05652-900 São Paulo, SP, Brazil; ^4^Division of Geriatrics, FMUSP, 01246-903 São Paulo, SP, Brazil; ^5^Department of Pathology, FMUSP, 01246-903 São Paulo, SP, Brazil; ^6^Department of Neurology and Pathology, University of California, San Francisco, CA 94143, USA

## Abstract

Parkinson's disease (PD)—classically characterized by severe loss of dopaminergic neurons in the substantia nigra pars compacta—has a caudal-rostral progression, beginning in the dorsal motor vagal nucleus and, in a less extent, in the olfactory system, progressing to the midbrain and eventually to the basal forebrain and the neocortex. About 90% of the cases are idiopathic. To study the molecular mechanisms involved in idiopathic PD we conducted a comparative study of transcriptional interaction networks in the dorsal motor vagal nucleus (VA), locus coeruleus (LC), and substantia nigra (SN) of idiopathic PD in Braak stages 4-5 (PD) and disease-free controls (CT) using postmortem samples. Gene coexpression networks (GCNs) for each brain region (patients and controls) were obtained to identify highly connected relevant genes (hubs) and densely interconnected gene sets (modules). GCN analyses showed differences in topology and module composition between CT and PD networks for each anatomic region. In CT networks, VA, LC, and SN hub modules are predominantly associated with neuroprotection and homeostasis in the ageing brain, whereas in the patient's group, for the three brain regions, hub modules are mostly related to stress response and neuron survival/degeneration mechanisms.

## 1. Introduction

Parkinson's disease (PD) is the second most common neurodegenerative disease worldwide [1]. Ageing is the main risk factor and about 90% of the cases are idiopathic [2]. PD features a substantial loss of neurons in the substantia nigra and locus coeruleus and systematic deposition of protein-rich aggregates in the brain as intracellular inclusions, forming the Lewy neurites and Lewy bodies [3]. *α*-Synuclein is the most abundant protein found in Lewy bodies and usually aggregates in fibrillar structures [4]. The disease displays a caudal-rostral progression, starting in the dorsal motor vagal nucleus and, in a less extent, in the olfactory system, progressing to the limbic structures and up to the neocortex [3, 5, 6]. This progression pathway is the basis of the widely accepted Braak staging model of PD [3, 5, 6]. Recent studies have shown that misfolded *α*-synuclein can be transferred between neurons in a prion-like manner and following the caudo-rostral progression pathway of Braak model [for revisions see [4, 7, 8]].

To better understand the molecular mechanisms underlying idiopathic PD, several studies compared global gene expression in postmortem samples (mostly in substantia nigra) from patients with PD and matched controls [9, 10]. Global gene expression is a functional genomic approach based on data derived from DNA microarray technology and analyzed by bioinformatics tools [11]. Typically, these studies were focused on the identification of differentially expressed genes and of genes involved in particular molecular pathways [9, 10]. Bioinformatics analysis was sometimes restricted to gene categorization [11]. A meta-analysis of 11 out 22 functional genomic studies conducted between 2004 and 2009 [10] failed to show a gene with reliable differential expression [9]. Nevertheless, six among nine studies reported deregulation in the metabolic pathways related to mitochondrial function/electron transportation, protein degradation, and synaptic transmission (only three reported alterations in dopamine signaling pathway) [9].

This scenario started to change with the introduction of statistical and computational tools for analyzing gene-gene interaction networks and the comparative analysis of gene expression and interactome [12]. Edwards et al. [13] used a typical systems biology approach to combine data from genome wide association studies (GWAS) and of gene expression in the six adjacent brain regions used for PD Braak staging. They found that calcium signaling, focal adhesion, and axonal guidance were the main consensus disease pathways in PD. Marei et al. [14] analyzing expression microarray data obtained from adult postmortem SN found that the genes enriched in SN cells included the following functional categories: synaptic transmission, central nervous system development, structural components of myelin sheath, internode region of axons, ion transport, and voltage-gated ion channel complex.

The relevant genes to the pathogenesis of complex diseases, like idiopathic PD, are those with a high number of gene-gene links in transcriptional interaction networks, which do not necessary show what a high differential expression [15, 16]. These highly connected genes, or hubs, are called “broker genes” in the sense that they connect many genes that would not be connected otherwise [15]. Therefore, gene coexpression network (GCN) studies may help to unravel molecular mechanisms in neurological diseases by offering genome-scale information. In fact, many recent investigations have shown that modular transcriptional repertoires, that is, communities of highly connected genes [17, 18], relate to fundamental features of brain activity and structure [19, 20]. Here, through DNA microarray gene expression data, we performed comparative analyses of gene coexpression networks (GCNs) in dorsal motor vagal nucleus (VA), locus coeruleus (LC), and substantia nigra (SN) of idiopathic Parkinson's disease patients at Braak stages 4-5 and matched controls using postmortem samples.

## 2. Materials and Methods

Postmortem samples of dorsal motor vagal nucleus (VA), locus coeruleus (LC), and substantia nigra (SN) from controls and PD subjects (Table 1) were obtained from the Brain Bank of the Brazilian Aging Brain Study Group, BEHEEC-FMUSP, under institutional (FMUSP) ethical committee approval 04/285 [21]. The samples (3-4 mm^3^) were homogenized with Tissue Rupter (Qiagen, catalog number 9001272, Valencia, CA), and total RNA was extracted from the homogenates using the RNeasy Lipid Tissue Kit (Qiagen, catalog number 74804, Valencia, CA). RNA quality was assessed on the Agilent BioAnalyzer 2100 (Agilent, Santa Clara, CA). RNA integrity number (RIN) values were all within the acceptable range (6-7) for microarray assays using brain bank samples [22]. To determine gene expression profiles, 44 K DNA microarrays (Agilent Technologies, catalog number G4845A, Santa Clara, CA) were used. The procedures for hybridization followed the protocols provided by the manufacturer's instructions (One-Color Microarray-Based Gene Expression Analysis, Quick Amp Labeling). The images were captured by the reader Agilent Bundle according to the parameters recommended for BioArrays and extracted by Agilent Feature Extraction software version 9.5.3. Among the 45,015 spots present in each array only those with none or only one flag (i.e., low intensity, saturation, controls, etc.) were selected for analysis using the R software version 2.11.1 (R Development Core Team, 2010) and the Lowess test for normalization. We identified 17,142 valid transcripts for SN samples (8 PD and 5 CT cases), 20,705 valid transcripts for LC samples (7 DP and 7 CT cases), and 18,681 valid transcripts for VA samples (8 DP and 6 CT cases). By means of the TMEV software version 4.6.1 [17] we selected differentially expressed transcripts (PD × CT). For LC samples, this comparison was performed using SAM (significance analysis of microarrays), whereas for SN and VA samples (nonparametric) Wilcoxon Mann-Whitney test (*P* < 0.005 or *P* < 0.01, resp.) was used. All microarray data were deposited in GEO public database (http://www.ncbi.nlm.nih.gov/geo) under accession number GE43490. Transcriptional interaction network for differentially expressed GO annotated genes was constructed based on Pearson's correlation, using software R. Data analysis and visualization were achieved through Cytoscape software 2.8.0 (http://www.cytoscape.org/).

## 3. Results and Discussion

The comparative analysis of VA, LC, and SN transcriptomic profiles for patient (PD) versus control (CT) groups revealed 234, 183, and 326 differentially expressed GO annotated genes, respectively. All genes were upregulated in PD groups. Transcriptional interaction networks for each anatomic region (VA, LC, and SN) for both groups were obtained and analyzed. In each of these six networks, the genes with the higher number of gene-gene links were considered hubs [21, 23] and the sets of highly interconnect genes were identified as modules [18–20].

## 4. VA Networks

**Figure 1:**
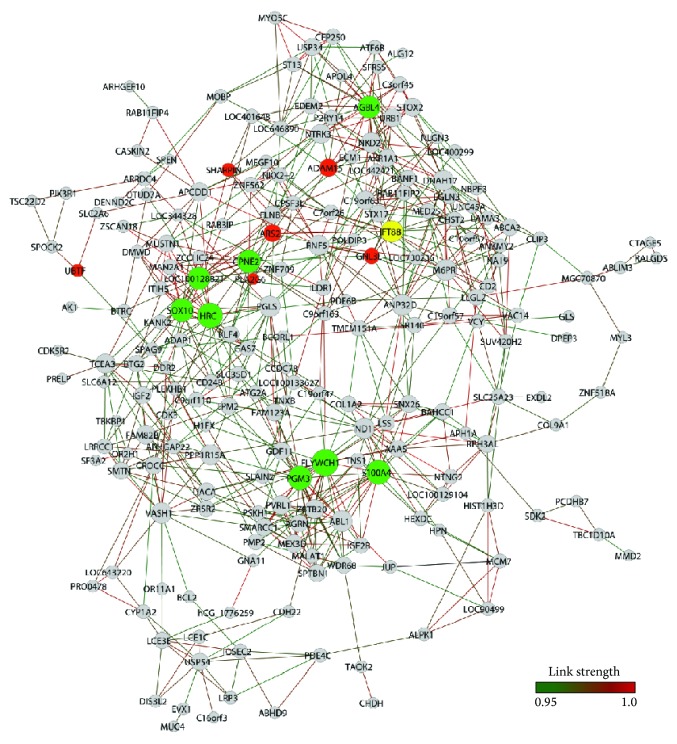
VA-CT gene expression network. Nodes in red indicate hubs of PD network, nodes in green indicate hubs of CT network, and node in yellow indicates a common hub of PD and CT networks.

**Figure 2:**
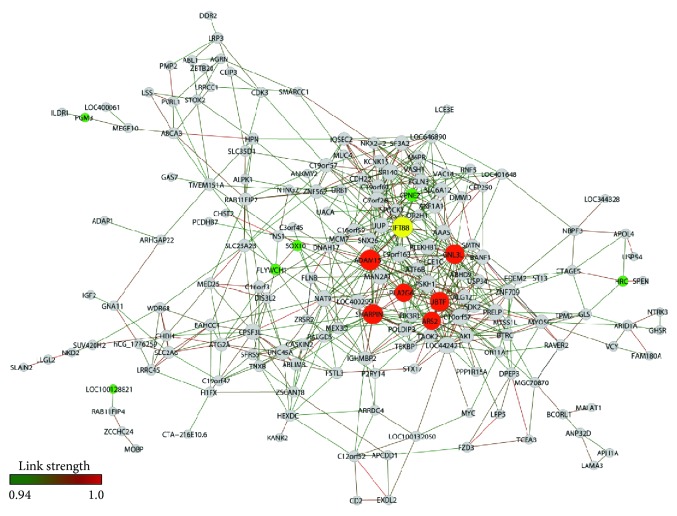
VA-PD gene expression network. Nodes in red indicate hubs of PD network, nodes in green indicate hubs of CT network, and node in yellow indicates a common hub of PD and CT networks.

A total of 178 genes and 646 gene-gene links (threshold 0.94) for VA-PD network and 206 genes and 670 gene-gene links (threshold 0.95) for VA-CT network were obtained, respectively (Figure 1 (VA-CT) and Figure 2 (VA-PD)). Network connectivity *k* for nondirected networks was calculated by *k* = 2*L*/*N*, where *L* stands for the number of edges and *N* for the number of nodes [24]. *k* values were 6.5 for VA-CT and 7.25 for VA-PD.  Table 2 lists the selected VA hubs of CT and PD groups.

The VA-CT network displayed a modular structure with four clearly identifiable modules (Figure 1). Two of these modules showed a sole central hub; one is centered in* AGBL4*, a gene involved in controlling polyglutamate side chains which is a critical process for neuron survival [33], and the other in* IFT88*, which is a common hub of both VA-CT and VA-PD networks.* IFT88* codes for a key component of intraflagellar transport were involved in dendrite patterning and synapse integration of adult-born neurons [34, 70, 71]. The other two modules encompass several highly linked hubs.

The first clusters were* CPNE2*,* HRC*,* SOX10,* and* ZEB2* (former LOC100128821).* CPNE2* and* HRC* are genes involved in brain Ca^2+^ metabolism and functions:* HRC,* or histidine-rich calcium binding protein, regulates Ca^2+^ homeostasis [26], whereas* CPNE2* acts as a Ca^2+^ sensor in postsynaptic events [32].* SOX10* and* ZEB2* play a role in myelination processes:* SOX10* codes for a transcription factor acting in regulating myelination in oligodendrocytes [31], and* ZEB2*, which codes for a Smad-interacting protein, acts in myelination of the central nervous system [72] and regulates the fate switch between cortical and striatal interneurons [73].

The second module harbored the genes* S100A4*,* PGM3,* and* FLYWCH1. S100A4* codes for a Ca^2+^-binding protein were involved in neuroprotection, rescuing neurons via the Janus kinase/STAT pathway and, partially, via interleukin-10 receptor [27], and promoting neuritogenesis and survival [28]. PGM3 codes for a phosphoglucomutase involved in glycogenesis and glycogenolysis; these processes provide energy for cellular calcium homeostasis [29] and cause hypomyelination when mutated [30].* FLYWCH1* codes a FLYWCH-type zinc finger 1 chromatin modulator protein. Cellular proteins that harbor the FLYWCH domain are predominantly involved in transcriptional regulation [25]. Altogether, the VA-CT modules encompass genes associated with neuron survival and protection, Ca^2+^ homeostasis, myelination, and neuron differentiation.

The VA-PD network (Figure 2) had, comparatively with VA-CT, a totally distinct topology and modular distribution. The highly connected hubs are all included in a single module. The majority of these genes take part in molecular and cellular processes related to stress responses.* ADAM15*, which codes for a disintegrin metalloprotease, has been implicated in both the process of neuronal hypoxic injury [35] and the protection (via GRP78 binding) of neurons from hypoxia-induced apoptosis [74].* SHARPIN* codes for a ubiquitin-binding and ubiquitin-like-domain-containing protein, which is an important component of the linear ubiquitin chain assembly complex (LUBAC) that modulates activation of NF-*κ*B signaling pathway, thus controlling cell survival and apoptosis [36–38].* GNL3* codes for a nucleolar protein which stabilizes* MDM2* (a nuclear-localized E3 ubiquitin ligase) in the nucleoplasm [39] and promotes neuronal survival [40].* UBTF* codes for a protein playing critical roles in ribosomal RNA transcription and chromatin remodeling, which takes part in the compensatory response to proteotoxic stress in neurons [41].* ARS2 *participates in maintaining neuronal stem cell identity via direct transcriptional activation of* Sox2* [42]. Finally,* PLA2G6*, aliase* PARK14*, codes for a phospholipase A2, group 6 which hydrolases membrane phospholipids and may contribute, via lipid peroxidation, to CNS injury and disorders, such as Parkinson's disease [43]. Not surprisingly, mutations in this gene cause an autosome recessive early-onset form of Parkinson's disease with widespread Lewy bodies [44].

## 5. LC Networks

**Figure 3:**
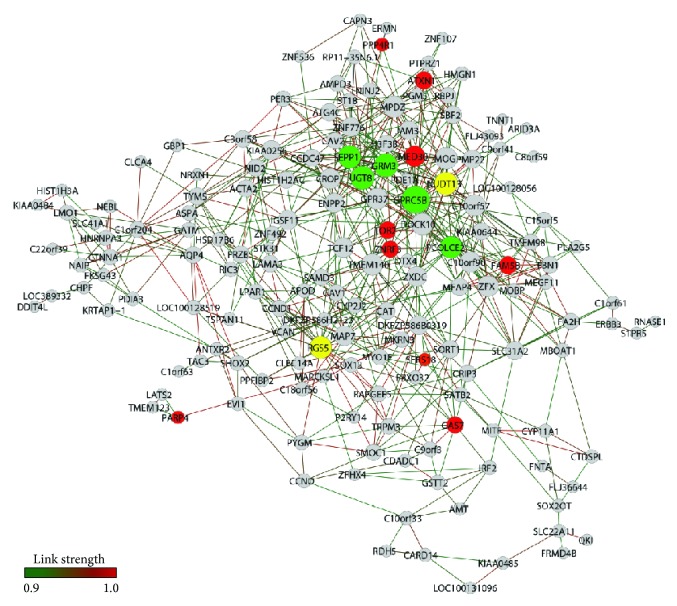
LC-CT gene expression network. Nodes in red indicate hubs of PD network, nodes in green indicate hubs of CT network, and nodes in yellow indicate common hubs of PD and CT networks.

**Figure 4:**
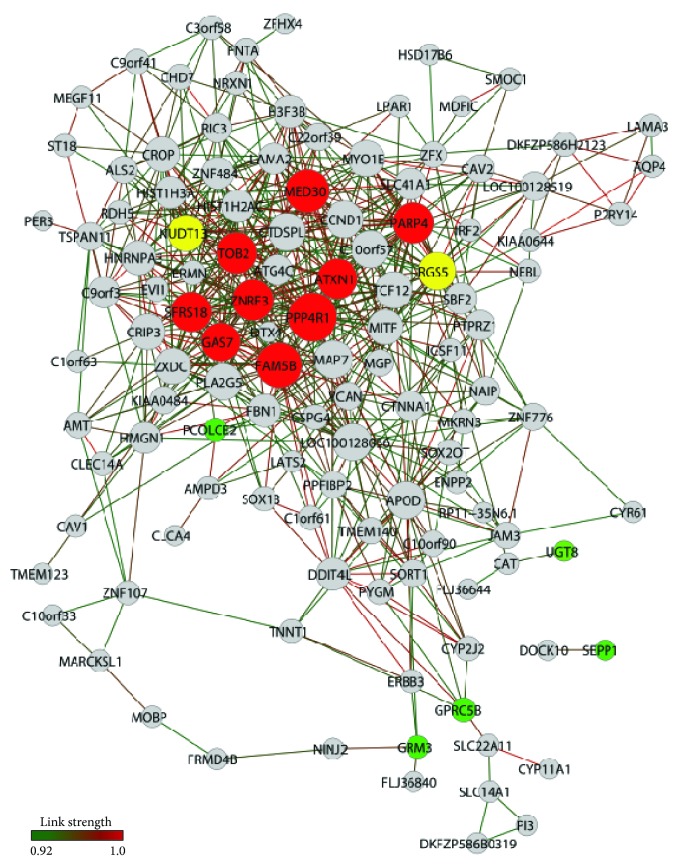
LC-PD gene expression network. Nodes in red indicate hubs of PD network, nodes in green indicate hubs of CT network, and nodes in yellow indicate common hub of PD and CT networks.

A total of 121 genes and 659 gene-gene links (threshold 0.92) and 164 genes and 645 gene-gene links (threshold 0.90) were obtained for LC-PD network and LC-CT network, respectively. *k* values were 7.86 and 10.89 for LC-CT (Figure 3) and LC-PD (Figure 4).  Table 3 lists the selected LC hubs for CT and PD groups.

In the LC-CT network the majority of the highly connected hubs were clustered in one large module encompassing several genes related to neuroprotection and maintenance of myelinated fibers in the aging brain (Figure 3). In this large module, two genes play a role in response to oxidative stress and antioxidant protection:* NUDT13*, a common hub of both LC-CT and LC-PD networks, facilitates the elimination of oxidized forms of NAD(P)H and CoA cofactors from peroxisomes, mitochondria, and the cytoplasm [51], whereas* SEPP1* codes for a brain antioxidant selenoprotein secreted by astrocytes and taken up by neurons via the apolipoprotein E receptor 2 [52].* GRM3*, the metabotropic glutamate receptor 3 gene, and* UGT8*, UDP-glucuronosyltransferase 8, are closely interconnected hubs of the LC-CT network (Figure 3) and exert important protective roles for the aging brain.* GMR3* is expressed by glia and neurons in many brain regions [46] and acts not only in glutamate transmission but also in the establishment and maintenance of myelinated fibers [47] and protection against mitochondrial neurotoxins [48].* UGT8* is involved in oligodendrocyte differentiation [49] and myelination processes [50].* GPR5B* codes for a G protein-coupled receptor, which is a member of the group C metabotropic glutamate receptor family. This protein is required for neuronal fate determination in the brain [45] and* GPR5B* downregulation affects microglial activation [75]. Directly linked to* GPR5B *(Figure 3) appears* PCOLCE2*, a gene coding for a procollagen C-proteinase enhancer [54] and involved in the regulation of proapolipoprotein (apo) AI (apoAI) posttranslational processing [55]: apoAI binds *β*-amyloid peptide, a major protein in the brain associated with Alzheimer's and Parkinson's diseases, thus preventing Abeta-induced neurotoxicity [76].

Lastly, a relatively small module was organized around* RGS5* (Figure 3), a regulator of G protein signaling and a well-known marker for brain pericytes [53, 77]. Pericytes contribute to the control of endothelial tight-junction cells and blood-brain barrier (BBB) function. There is a correlation between BBB dysfunction and the progression of Parkinson's disease [78]. Interestingly,* RGS5* is a common hub of LC-CT and LC-PD networks.

In the LC-PD network all highly linked hubs are clustered in a single central module (Figure 4). Inside this module, eight out eleven genes are associated with neuroprotection and brain homeostasis. Two of these genes,* NUDT13* and* RGS5*, were common hubs of LC-CT network and their roles have been already described. Two other genes,* PPP4R1* which codes for a protein phosphatase 4 catalytic unity [56] and* ATXN1* which codes for a polyglutamine-containing protein (polyQ) and may cause neurodegenerative diseases depending on the length of polyQ expansions [65], modulate transcriptional repression through binding to histone deacetylase 3 [79, 80]. Transcriptional repression is an important epigenetic mechanism controlling the expression of essential genes for neuron survival and its imbalance may cause PD [81].

The remaining four genes in this set also exert relevant functions in neuroprotection and brain homeostasis, some of them possibly linked to repairing cellular injuries in PD, as discussed below.

The gene* MED30*, which codes for the mediator of polymerase II transcription subunit 30, participates in oxidative phosphorylation and mitochondrial integrity [58].* TOB2* regulates mRNA deacetylation, potentiates NGF-induced differentiation, and protects neurons from apoptosis [59, 60].* SFRS18* (aliases* SF2*/*AFS*) codes for a serine-arginine rich protein which regulates protein sumoylation [66], a process required for inhibiting *α*-synuclein aggregation and toxicity [67]. Finally,* ZNFR3*, a gene coding for a cell surface transmembrane E3 ubiquitin ligase zinc and ring finger 3, promotes Wnt receptor turnover [61]. This gene is critical because Wnt signaling is linked to (i) synaptic maintenance in the adult aging brain [62], (ii) regulation of inflammatory pathways along PD progression [63], and (iii) differentiation of LC noradrenergic neuronal precursors [104]. Recent findings indicate that dysregulation of the crosstalk between Wnt/*β*-catenin signaling and antioxidant/anti-inflammatory pathways leads to the decline of subventricular zone (SVZ) plasticity with age and the limited nigrostriatal dopaminergic self-repair in PD [63]. Therefore, further investigation on the role of* ZNFR3* in PD would be necessary.

Furthermore, inside the main hub cluster (Figure 4) three genes can be related to repair functions in PD: the growth arrest-specific gene 7, or* GAS7*, which promotes neurite outgrowth and motor neuron function [68, 69],* FAM5B*, alias* BRIMP2*, which codes for a BMP/RA-induced neural protein widely expressed in the central nervous system and related to neuron growth [57], and* PARP4*, which codes for a poly-ADP-ribose polymerase involved in the control of synaptic plasticity via major vault protein [64].

Therefore, in LC-PD there is an important activity of genes that could exert repair or compensatory mechanisms. In PD, the compensatory mechanisms at cellular and molecular levels are centered in protection against neurotoxicity [105, 106] and neurogenesis and reinnervation of affected areas [107]. These mechanisms are more active in the initial and intermediate stages of PD, declining in the final stages [108, 109].

## 6. SN Networks

**Figure 5:**
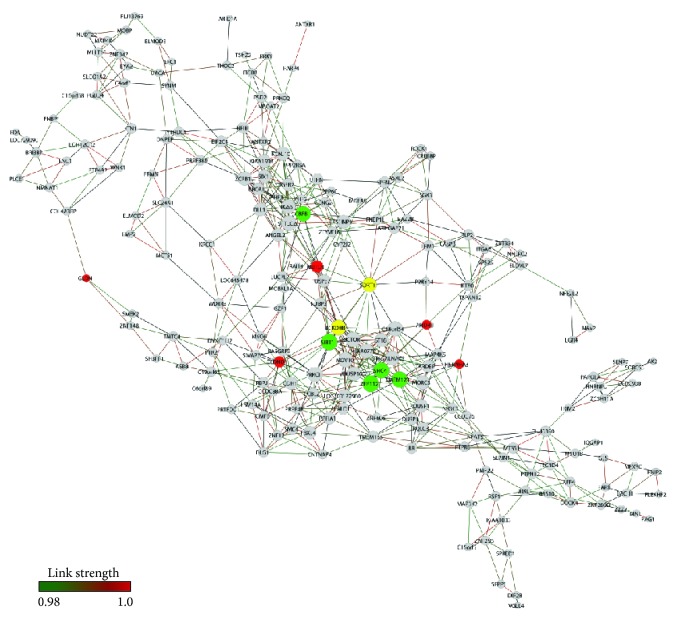
SN-CT gene expression network. Nodes in red indicate hubs of PD network, nodes in green indicate hubs of CT network, and nodes in yellow indicate common hubs of PD and CT networks.

**Figure 6:**
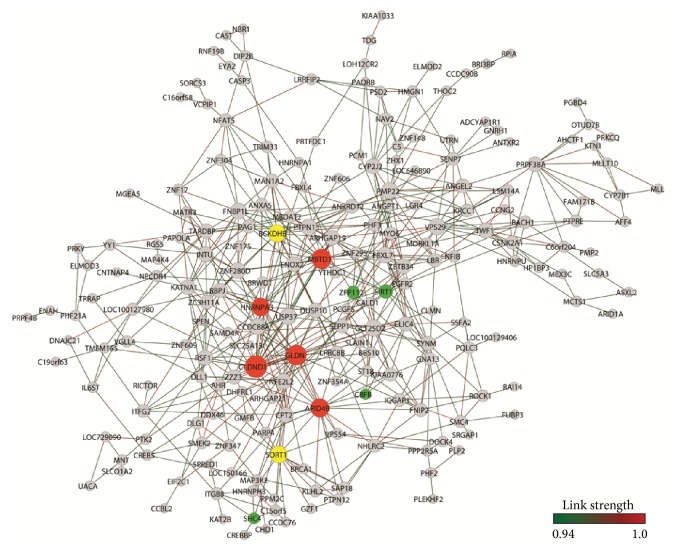
SN-PD gene expression network. Nodes in red indicate hubs of PD network, nodes in green indicate hubs of CT network, and nodes in yellow indicate common hubs of PD and CT networks.

A total of 209 genes and 586 gene-gene links (threshold 0.94) and 199 genes and 682 gene-gene links (threshold 0.97) were obtained for SN-PD network and SN-CT network, respectively. *k* values were 6.85 and 5.60 for SN-CT and SN-PD.  Figure 5 depicts SN-CT and Figure 6 depicts SN-PD.  Table 4 lists the selected SN hubs of CT and PD groups.

In the control group network (SN-CT), the highly linked hubs were clustered in two modules (Figure 5). One module has* CBFB* as a center, a gene coding for the beta subunit of a core-binding transcription factor belonging to the PEBP2/CBF transcription factor family which controls the transcriptional regulation of neurotrophin receptors, some ion channels, and neuropeptides, playing important roles in neuron development [91, 92]. The other module encompassed the remaining six SN-CT hubs.

Two hubs,* BCKDHB* and* SIRT1*, were closely linked (Figure 5) and play significant roles in neuroprotection and brain homeostasis.* BCKDHB*, which is a common hub of SN-CT and SN-PD networks, codes for a branched-chain keto acid dehydrogenase E1 beta polypeptide, a multienzyme complex associated with the inner membrane of mitochondria that acts in the catabolism of branched-chain amino acids and is essential for glutathione homeostasis [89, 90].* SIRT1* codes for a NAD-dependent deacetylase (sirtuin 1) and play important neuroprotective roles in the aging brain and PD [82].* SIRT1* deacetylates heat shock factor 1 (HSF1), increasing the transcription of molecular chaperones such as heat shock protein 70.* SIRT1* interacts with peroxisome proliferator-activated receptor-gamma coactivator-1*α* (PGC-1*α*) to reduce oxidative stress and increase the viability of dopaminergic neurons [83]. Moreover,* SIRT1* may also regulate autophagy and mitophagy, which may diminish *α*-synuclein toxicity in PD [110]. Interestingly, these two genes interact directly with* SORT1* (sortilin1), a common hub of SN-CT and SN-PD networks. Sortilin is a member of the family of vacuolar protein sorting 10 protein domain receptors. It is a coreceptor in cell death and neurodegeneration processes mediated by proneurotrophins (proNT) [101, 102] and it might contribute to neuronal apoptosis or neurodegeneration during pathogenesis and disease progression of Parkinson's disease [103]. It is worth to note that proNT-mediated cell death is also relevant in normal development and during senescence of the nervous system:* SIRT1* expression is altered in the aging brain and aged neurons are more sensitive to proNT-induced killing than young ones [111]. Consequently, sortilin may contribute to brain's functional integrity during normal physiological conditions.

Three other hubs are* ZFP112*, a gene coding for an unknown zinc-finger protein, and* SHC4* and* TMEM123*, both having relevant roles in neuronal homeostasis.* SHC4* codes for a Src homology and collagen (Shc) protein that interacts with tropomyosin receptor kinase B, trkB [84], the high affinity receptor for BDNF expressed in striatal neurons. BNDF can reverse neuronal injury associated with PD [85].* TMEM123* codes for transmembrane protein 123, a cell surface receptor mediating oncolytic cell death [86]. Oncosis may be triggered by oxidative stress [87], and it is important for neuronal homeostasis [88]. Altogether, the SN-CT network presented a situation compatible with SN functioning in the aging brain.

Conversely, the SN-DP network showed a significant change in the modular structure with all the highly connected hubs clustered in a single module (Figure 6). Two of these hubs,* BCKDHB* and* SORT1* (previously discussed), are common to both SN-CT and SN-PD networks. The most connected hub in SN-PD network (Table 4) was* CLDN1*, a gene which codes for claudin 1, a protein expressed in substantia nigra pars compacta [93] and involved in tight-junction formation at the BBB [78]. In fact, BBB dysfunction occurs in many neurodegenerative diseases, such as PD [78]. The second most connect hub is* GLDN*, a gene coding for gliomedin, a molecule well known for mediating heterotypic cell-cell adhesion and interacting with neurofascin-186 (NF186) and neuronal cell adhesion molecule (NrCAM) in the molecular assembly of the nodes of Ranvier in the peripheral nervous system [94, 95]. Since functional genomic studies show that dopaminergic neurons in adult human SN support, or modulate, myelin sheath formation and voltage-gated ion channel activity [14], it is reasonable to assume that* GLDN* expressed in SN cells could have a role in these processes. Furthermore, diffusion tensor imaging studies showed significant regional (substantia nigra) and global white matter deterioration in PD [112].

The last three SN-PD hubs,* ARID4B, MBTD1, *and* HNRNPA3,* have been shown to participate in biological processes associated with neurodegeneration.* ARID4B* is a chromatin remodeling gene coding for a protein associated with the mSIN3A histone deacetylase (HDAC) complex [98], which participates in neuronal apoptosis and transcriptional signaling in neurodegenerative diseases [99].* MBTD1 *is a member of the Polycomb gene family [113], and its protein product binds the Rb- (retinoblastoma-) E2F complex [96], thus contributing to cell cycle progress and apoptosis [97]. In PD, the Rb-E2F pathway activates mitosis-like signals in dopaminergic neurons of SN pars compacta mediating the death of these cells [114].* HNRNPA3 *codes for a shuttling RNA transporter found in the neuronal RNA granules and P-bodies [100]. These structures are associated with altered ribostasis, dendrite sprouting, and neurodegeneration [115, 116]. Here is important to note that* SIRT1*, a SN-CT hub, exerts its neuroprotective action by inhibiting the* ARID4B*/mSINA3/HDAC transcriptional repression activity [117]. This* SIRT1* activity gets apparently attenuated in the SN-PD network.

The SN-PD transcriptional network profile, with a predominance of hubs linked to neurodegenerative processes (although retaining one hub involved in glutathione homeostasis and another in response to oxidative stress, both common to SN-CT), would be expected to be found in the SN of patients in Braak stages 4-5 [3, 8, 118–120].

Solid evidences now exist that PD has a caudal-rostral progression, being initiated in the dorsal motor vagal nucleus and/or olfactory bulb, progressing to the midbrain and eventually to the basal forebrain and the neocortex [3, 5, 6] through a prion-like mechanism of neuron-neuron transfer of altered *α*-synuclein molecules [4, 8, 121]. The SN, affected by Lewy body pathology in the Braak stages 3-4 of PD [3, 5, 6], showed in this study (Braak stages 4-5), diminished activation of genic circuits linked to neuroprotection when compared to the genomic profiles in VA and LC. Diminished activation is in agreement with the well-established fact that clinical diagnosis of PD can be made from Braak stage 3 onwards, although patients may present prodromal signs in early stages [119, 121]. It is worth to note that even in Braak stages 4-5 the genes linked to neuroprotection display high connectivity in VA and LC networks, what indicates that these genes are actively coordinating their particular cellular processes [17].

## 7. Conclusions

This report shows that transcriptional interaction network analysis, an effective methodology for dealing with a large set of genomic data [17, 19, 20], allowed the comparative study of transcriptome signatures in VA, LC, and SN in PD. These analyses identified the highly connected hubs and hub modules that possibly play relevant roles in the brain aging and/or PD progression. Accordingly, the comparative analysis between hub profiles in different anatomic regions for PD patients and controls revealed interesting scenario.

In VA-CT, the main hubs are associated with Ca^2+^ homeostasis, myelination, and neuroprotection in the aging brain, whereas, in VA-PD, the relevant hubs are mostly related to compensatory responses to proteotoxic stress. Interestingly, one of these hubs is* PARK14* (alias* PLA2G6*), a gene causing autosome recessive early-onset PD when mutated [44]. The LC-CT hubs are mainly associated with protection against oxidative and proteotoxic stress, myelination, and BBB maintenance. The majority of LC-PD hubs are also linked to neuroprotection and brain homeostasis, although in the context of repairing/compensating various PD-associated cellular injuries (*SFRS18*,* ZFNR3,* and* FAM5B*, for instance). Finally, the SN-CT main hubs include genes that are critical for neuroprotection and homeostasis in the aging brain, such as* SIRT1* and* BCKDHB*. Conversely, the SN-PD network displays a very different landscape: six out seven hubs are associated with neurodegenerative processes. These results are compatible with the caudo-rostral model of PD progression and point out to the usefulness of GCNs approach for (i) investigating the molecular mechanisms underlying idiopathic PD and (ii) identifying novel therapeutic targets based on the concept of interventions aimed to restore altered regulatory network structures [19, 20, 122].

## Figures and Tables

**Table 1:** Pathological data of patients and controls.

ID patients (PD) and controls (CT)	Braak staging	Gender	Age (yrs)	Anatomic region^*^		
				SN	LC	VA
PD1	4	F	85	X		X
PD2	4	M	64	X	X	X
PD3	4	M	66	X	X	X
PD4	4	F	80	X	X	X
PD5	4	F	84	X	X	X
PD6	5	M	68	X	X	X
PD7	5	M	82	X	X	X
PD8	5	M	90	X	X	X
CT1	Control	M	69		X	
CT2	Control	F	90		X	X
CT3	Control	M	64	X	X	X
CT4	Control	M	70	X	X	X
CT5	Control	M	85	X	X	X
CT6	Control	M	58	X	X	X
CT7	Control	F	70	X	X	X

F: female; M: male; SN: substantia nigra; LC: locus coeruleus; VA: dorsal motor vagal nucleus; ^*^genomic study.

**Table 2:** Main hubs in VA-CT and VA-PD networks^*^.

Gene	Gene-gene links		Gene product and/or biological function
	CT	PD	
FLYWCH1	**23**	6	FLYWCH-type zinc finger 1. DNA binding. Involved in transcriptional regulation [25]
HRC	**21**	4	Histidine rich calcium binding protein. Regulator of Ca^2+^ homeostasis [26]
S100A4	**20**	0	S100A4 Ca^2+^-binding protein involved in neuroprotection. It rescues neurons via the Janus kinase/STAT pathway and, partially, the interleukin-10 receptor [27, 28]
PGM3	**19**	2	Phosphoglucomutase 3. PGM3 is involved in glycogenolysis and glycogenesis; these processes provide metabolic energy for cellular calcium homeostasis [29] and causes hypomyelination when mutated [30]
SOX10	**18**	6	Transcription factor. Involved in regulatory network for myelination in oligodendrocytes [31]
CPNE2	**18**	0	Calcium-dependent membrane binding protein. Ca^2+^ sensor in postsynaptic events [32]
LOC100128821	**17**	1	Hypothetical protein LOC100128821
AGBL4	**17**	0	ATP/GTP binding protein-like 4. CCP6 (aliase). CCP6 catalyzes the shortening of the glutamate side chains, a critical process for neuron survival [33]
IFT88	**16**	**26**	Key component of intraflagellar transport and involved in neuron migration and dendrite arborization [34]
ADAM15	11	**26**	Metalloprotease-disintegrin expressed in brain and involved in neuroprotection [35]
SHARPIN	5	**25**	Ubiquitin-binding and ubiquitin-like-domain-containing protein. It modulates activation of NF-*κ*B signaling pathway and controls cell survival and apoptosis [36–38]
GNL3L	8	**24**	Guanine nucleotide binding protein-like 3 nucleolar-like, paralogue of nucleostemin (NS). GNL3L, as NS, stabilizes MDM2 protein promoting neuron survival [39, 40]
UBTF	3	**24**	Upstream binding transcription factor, RNA polymerase I (aliase UBF), is a transcriptional activator regulating rRNA transcription. The activation of the nucleolar transcription is a response to proteotoxic stress in neurons [41]
ARS2	10	**21**	Ars2 maintains neural stem cell identity via direct transcriptional activation of Sox2 [42]
PLA2G6	2	**21**	Phospholipase A2, group VI (cytosolic, calcium-independent), PARK14 (aliase). PARK14 gene encodes iPLA2-VIA, a calcium independent phosphatase, catalyzing the hydrolysis of glycerophospholipids. Mutations in this gene can cause autosome recessive early-onset form of PD [43, 44]

^*^Bold numbers indicate highly linked hubs in CT and/or PD networks.

**Table 3:** Main hubs in LC-CT and LC-PD networks^*^.

Gene	Gene-gene links		Gene product and/or biological function
	CT	PD	
GPRC5B	**30**	6	Orphan G protein-coupled receptor (putative glutamate receptor candidate) required for neuronal differentiation [45]
GRM3	**23**	4	Group II metabotropic glutamate receptor modulating glutamate neurotransmission and synaptic plasticity. It plays a role in neuroprotection and white matter integrity [46–48]
UGT8	**23**	1	UDP glycosyltransferase 8. Highly expressed in brain oligodendrocytes. Involved in myelination and maintenance of white matter tracts within the central nervous system [49, 50]
NUDT13	**21**	23	Mitochondrial enzyme (Nudix hydrolase) involved in response to oxidative stress [51]
SEPP1	**21**	1	Selenoprotein P, plasma, 1. Maintains selenium homeostasis in the brain. Involved in antioxidant protection of astrocytes and neurons [52]
RGS5	**20**	**25**	Regulator of G protein signaling 5. It is a marker of brain pericytes [53]
PCOLCE2	**20**	5	Procollagen C-endopeptidase enhancer 2 [54]. It regulates apoAI posttranslational processing [55]
PPP4R1	4	**40**	Protein phosphatase 4 catalytic unit [56]
FAM5B	12	**35**	BRINP2 (aliase). BMP/RA-inducible neural specific protein. BRINP1, BRINP2, and BRINP3 are predominantly and widely expressed in both the central nervous system (CNS) and the peripheral nervous system (PNS) and involved in neuron development [57]
MED30	17	**32**	Mediator of RNA polymerase II transcription subunit 30. Required for oxidative phosphorylation and mitochondrial integrity [58]
TOB2	11	**29**	TOB2 regulates mRNA deadenylation, potentiates NGF-induced differentiation, and protects neurons from apoptosis [59, 60]
ZNRF3	11	**29**	ZNRF3 promotes Wnt receptor turnover [61]. Wnt signaling is linked to synaptic maintenance in the aging brain [62] and regulation of inflammatory pathways along PD progression [63]
PARP4	3	**29**	Poly-ADP-ribose polymerase controlling synaptic plasticity via major vault protein [64]
ATXN1	12	**28**	Polyglutamine-containing protein. Polyglutamine (polyQ) disease gene putatively involved in autosomal dominant Parkinson's disease [65]
SFRS18	3	**28**	Serine-arginine rich protein (aliase SF2/AFS) regulates protein sumoylation [66]. Protein sumoylation inhibits alfa-synuclein aggregation and toxicity [67]
GAS7	10	**26**	Growth arrest-specific gene 7 (Gas7) is involved in neurite outgrowth and motor neuron function [68, 69]

^*^Bold numbers indicate highly linked hubs in CT and or PD networks.

**Table 4:** Main hubs in SN-CT and SN-PD networks^*^.

Gene	Gene-gene links		Gene product and/or biological function
	CT	PD	
SIRT1	**19**	10	Nicotinamide adenine dinucleotide- (NAD+-) dependent deacetylase (sirtuin 1) promotes axonal elongation, neurite outgrowth, and dendritic branching. Sirtuin 1 plays a relevant protective role in PD [82, 83]
ZFP112	**19**	8	Zinc finger protein 112 homolog
SHC4	**18**	5	SHC (Src homology 2 domain containing) family, member 4 (aliase ShcD), interacts with tropomyosin receptor kinase B, trkB [84], the high affinity receptor for BDNF expressed in striatal neurons. BNDF can reverse neuronal injury associated with PD [85]
TMEM123	**18**	0	Transmembrane protein 123, a cell surface receptor mediating oncotic cell death [86]. Oncosis may be triggered by oxidative stress [87] and is important for neuronal homeostasis [88]
BCKDHB	**17**	**17**	Branched chain keto acid dehydrogenase E1, beta polypeptide, a multienzyme complex associated with the inner membrane of mitochondria, functioning in the catabolism of branched chain amino acids. BCKDHB is essential for glutathione homeostasis [89, 90]
CBFB	**17**	4	Core-binding factor, beta subunit, is a transcription factor that plays critical roles in neuron development [91, 92]
CLDND1	9	**26**	Claudin 1. Claudin-family proteins are involved in tight-junction formation at the blood-brain barrier (Luissint et al., 2012 [78]) and CLDN1 is expressed in the substantia nigra compacta [93]
GLDN	3	**22**	Gliomedin (Gldn) secreted by Schwann cell microvilli binds NgCAM-related CAM (NrCAM) and neurofascin-186 (NF186); it mediates heterotypic cell-cell adhesion [94, 95]
MBTD1	11	**21**	Member of the Polycomb group (PcG) protein family. It binds the Rb-E2F complex and contributes to cell cycle progress and apoptosis [96, 97]
ARID4B	6	**19**	Chromatin remodeling gene coding for a protein associated with mSIN3A histone deacetylase complex [98]. It is involved in neuronal apoptosis [99]
HNRNPA3	6	**18**	Shuttling RNA transporter found in neuronal RNA granules and P-bodies [100]
SORT1	15	**16**	Sortilin 1 is a member of the family of vacuolar protein sorting 10 protein domain receptors. It is a coreceptor in cell death and neurodegeneration processes mediated by proneurotrophins [101, 102]; it contributes to neuronal apoptosis or neurodegeneration during pathogenesis and progression of Parkinson's disease [103].

^*^Bold numbers indicate highly linked hubs in CT and or PD networks.
